# The Cellular and Physiological Functions of the Lowe Syndrome Protein OCRL1

**DOI:** 10.1111/tra.12160

**Published:** 2014-03-07

**Authors:** Zenobia B Mehta, Grzegorz Pietka, Martin Lowe

**Affiliations:** 1 Faculty of Life Sciences, University of ManchesterThe Michael Smith Building, Oxford Road, Manchester, M13 9PT, UK; 2 Current address: Faculty of Medicine, Imperial CollegeLondon, UK; 3 Current address: Cancer Institute, Faculty of Medical SciencesUCL, London, UK

**Keywords:** actin, ciliogenesis, Dent disease, endocytosis, inositol 5-phosphatase, OCRL1, oculocerebrorenal syndrome of Lowe, phagocytosis, phosphoinositide, zebrafish

## Abstract

Phosphoinositide lipids play a key role in cellular physiology, participating in a wide array of cellular processes. Consequently, mutation of phosphoinositide-metabolizing enzymes is responsible for a growing number of diseases in humans. Two related disorders, oculocerebrorenal syndrome of Lowe (OCRL) and Dent-2 disease, are caused by mutation of the inositol 5-phosphatase OCRL1. Here, we review recent advances in our understanding of OCRL1 function. OCRL1 appears to regulate many processes within the cell, most of which depend upon coordination of membrane dynamics with remodeling of the actin cytoskeleton. Recently developed animal models have managed to recapitulate features of Lowe syndrome and Dent-2 disease, and revealed new insights into the underlying mechanisms of these disorders. The continued use of both cell-based approaches and animal models will be key to fully unraveling OCRL1 function, how its loss leads to disease and, importantly, the development of therapeutics to treat patients.

## Overview of Phosphoinositides

Phosphoinositides are membrane phospholipids that regulate diverse cellular processes including gene expression, cytokinesis, cell motility, actin cytoskeleton remodeling, membrane trafficking and cell signaling 1,2. There are seven phosphoinositide species found in nature, generated by reversible phosphorylation of phosphatidylinositol at the 3′-, 4′- and/or 5′-positions of the inositol ring. Conversion between the different phosphoinositide species is mediated by specific kinases and phosphatases, of which there are more than 50 in vertebrates 3. Although extremely important, phosphoinositides comprise less than 1% of total cellular phospholipids, with PtdIns4P and PtdIns(4,5)P_2_ being the most abundant.

Phosphoinositides regulate cellular processes through direct interaction with effector proteins, which is mediated by phosphoinositide-binding domains found in these proteins 4,5. The best-described phosphoinositide-binding domains are the PH (pleckstrin homology), PX (phox homology), FYVE (Fab1, YOTB, Vac1 and EEA1), ENTH (epsin amino-terminal homology) and FERM (band 4.1, ezrin, radixin and moesin) domains. Of these, the PH domain is the most promiscuous, binding to PtdIns3P, PtdIns4P, PtdIns(3,4)P_2_, PtdIns(4,5)P_2_ and PtdIns(3,4,5)P_3_. Binding to effectors most typically leads to effector recruitment to the membrane. However, as exemplified by the PH domain, binding of an effector protein to a particular phosphoinositide can be inherently weak or lack specificity 6. In such cases, the fidelity of effector recruitment is conferred by additional interactions with the membrane, often in the shape of a protein partner, such as a small GTPase. This type of targeting has been coined ‘coincidence detection’ 7. In addition to recruiting effectors to membrane surfaces, phosphoinositides can also induce conformational changes in effector proteins to alter their biological activity. Examples include the PtdIns(4,5)P_2_-dependent activation of ion channels at the plasma membrane 8, or the PtdIns(4,5)P_2_-mediated conversion of the actin nucleation-promoting factor N-WASP from a closed, autoinhibited state to an open, activated state 9.

Each phosphoinositide species has its own unique subcellular distribution and most organelles appear to be enriched in a specific phosphoinositide; for example, PtdIns(4,5)P_2_ is abundant at the plasma membrane, whereas PtdIns3P and PtdIns4P are enriched at early endosomes and the Golgi apparatus, respectively 1,10. Thus, phosphoinositides contribute to compartmental identity 11. Interestingly, the sequential conversion from one phosphoinositide species to another can lead to a switch in compartment identity, as seen in the maturation of compartments within the early endocytic pathway 12,13. Segregation of phosphoinositides to different membranes may also promote directionality of membrane traffic between distinct compartments 14. Although most compartments appear to be associated with a particular phosphoinositide, as would be expected if they impart compartment identity, there is evidence that more than one phosphoinositide can reside on the same compartment. For example, in addition to the Golgi apparatus, PtdIns4P is abundant at the plasma membrane 15, where it has functions distinct from that of PtdIns(4,5)P_2_ 16. In addition to PtdIns3P, pools of PtdIns4P, PtdIns(4,5)P_2_ and PtdIns(3,4,5)P_3_ have also been detected on endosomes 17–19. Moreover, PtdIns3P can also be generated at the plasma membrane or endoplasmic reticulum during insulin signaling or autophagy, respectively 20,21. It is likely that distinct phosphoinositides residing on the same compartment are tightly controlled both spatially and temporally to ensure the coordinated recruitment of downstream effector proteins to specific membrane domains.

## Phosphoinositide Kinases and Phosphatases

The generation and turnover of phosphoinositides is determined primarily by the activity of specific phosphoinositide kinases and phosphatases, which themselves are highly regulated 3. This regulation can occur at the level of expression, or through localized recruitment and/or activation of catalytic activity, which is often mediated through binding to small GTPases. Additionally, binding to scaffolding proteins can help recruit these enzymes, and post-translational modification may also regulate their activity. The various enzymes are classified based on substrate preference and conservation of sequence and predicted domains. In addition to phosphoinositide kinases and phosphatases, it is also worth bearing in mind that levels of PtdIns(4,5)P_2_ can be affected by phospholipase C 22. In response to various stimuli, this enzyme hydrolyzes PtdIns(4,5)P_2_ to generate the second messengers diacylglycerol (DAG) and Ins(1,4,5)P_3_ (IP_3_) that are responsible for protein kinase C activation and mobilization of intracellular calcium stores, respectively.

OCRL1, the focus of this review, is 1 of 10 inositol 5-phosphatases present in vertebrates 23. Of these, only one (INPP5A) acts on soluble inositol polyphosphates, whereas the remainder prefer the lipid substrates PtdIns(4,5)P_2_ and PtdIns(3,4,5)P_3_. These enzymes use a common mechanism to catalyze hydrolysis of the phosphate at the 5′-position of the inositol ring 24,25. The different 5-phosphatases are localized to distinct cell types or distinct subcellular compartments within the same cell, where they regulate the abundance and turnover of distinct phosphoinositide pools. Consequently, the 5-phosphatases are important for various cellular and physiological processes, and dysfunction of a number of 5-phosphatases is associated with human disease 23,26,27. For example, mutation of OCRL1 results in Lowe syndrome and Dent-2 disease, discussed in further detail below. Mutations in INPP5E lead to Joubert and MORM syndromes, ciliopathies associated with mental impairment and various other developmental defects, whereas mutation of synaptojanin 1 is associated with early onset Alzheimer's disease in Down's syndrome. In contrast, SHIP1 and SHIP2 have been linked to immunity, and cancer and metabolic syndrome, respectively.

## Lowe Syndrome and Dent-2 Disease

**Figure 1:**
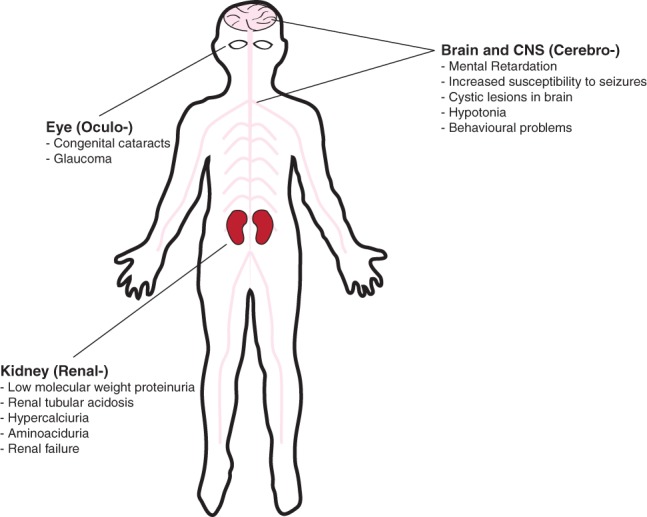
Schematic diagram showing the organs affected in Lowe syndrome. Oculocerebrorenal syndrome of Lowe affects the eyes, central nervous system and kidneys, with specific manifestations in each organ as indicated. Dent-2 affects the same organs and displays similar manifestations, although the ocular and neurological defects are typically milder than those seen in Lowe syndrome. Renal tubular acidosis is also less frequently observed in Dent-2 disease.

Mutation of OCRL1 was originally shown to be the cause of the rare X-linked disorder oculocerebrorenal syndrome of Lowe (OCRL), or Lowe syndrome, by Nussbaum and coworkers in 1992 28. More recently, patients diagnosed with a related X-linked disorder, called Dent disease, were also found to have mutations in OCRL1 29. This was surprising because Dent disease is typically caused by mutation of CLC5, an endosomal chloride/proton antiporter 30. Hence, Dent disease attributed to OCRL1 mutation has been named Dent-2 disease, with Dent-1 describing patients with CLC5 mutation 31. Both Lowe syndrome and Dent-2 disease are characterized by a selective proximal tubulopathy, caused by impairment of proximal tubular cells in the kidney 31,32. Symptoms include low-molecular-weight proteinuria, renal tubular acidosis, hypercalciuria and aminoaciduria (Figure 1). These symptoms can lead to progressive glomerular dysfunction, eventually resulting in renal failure. In addition to the renal symptoms, Lowe syndrome, and to a lesser extent Dent-2 disease, also causes defects in the eyes and nervous system 31,33. Ocular manifestations include congenital cataracts and glaucoma, while the neurological symptoms comprise hypotonia and mental retardation and an increased susceptibility to seizures. Magnetic resonance imaging has shown that some Lowe syndrome patients have white matter abnormalities, mainly cystic lesions in the periventricular region. The severity of Lowe syndrome and Dent-2 phenotypes varies widely between patients, even those carrying the same mutation in OCRL1, suggesting that genetic background is important, with genetic ‘modifiers’ determining the severity of the phenotype 34,35. Disease-causing mutations in OCRL1 typically result in loss of 5-phosphatase activity, or the absence of the protein itself due to loss of expression or degradation as a consequence of misfolding 35. Presently, it is not yet understood how loss of OCRL1 function leads to the symptoms associated with Lowe syndrome and Dent-2 disease.

This review will discuss recent progress on the cellular functions of OCRL1 and advances made using recently described animal models of Lowe syndrome and Dent-2 disease. Although we will briefly discuss OCRL1 domain organization and its various interaction partners, the focus of this review will be OCRL1 function, both at the cellular and organismal level. For a more detailed discussion of OCRL1 structure and physical interactions, we refer the reader to an excellent recent review by Pirruccello and De Camilli 25.

## Domain Organization and Interactions of OCRL1

OCRL1 is a multidomain protein with an N-terminal PH domain, a central 5-phosphatase domain and C-terminal ASH and RhoGAP-like domains 25. The PH domain is not evident from the amino acid sequence, and was discovered using 3D-NMR spectroscopy; it lacks the basic patch required for phosphoinositide binding and does not bind phosphoinositide-containing liposomes, but may be involved in interactions with other proteins 36. It also contains a loop outside of the domain fold with a clathrin-binding motif that helps recruit OCRL1 to endocytic clathrin-coated pits 36,37. The PH domain is connected to the 5-phosphatase domain via a flexible linker that contains an FEDNF motif responsible for binding the AP2 clathrin adaptor 38.

The structure of the catalytic 5-phosphatase domain of OCRL1 has not been determined. However, the structures of related 5-phosphatases, including the closest homolog of OCRL1, INPP5B, have been determined 24,25. Consequently, we have a good appreciation of the catalytic mechanism, which is similar to that used by Mg^2+^-dependent nucleases, and the residues that contribute to the domain fold. OCRL1 preferentially dephosphorylates PtdIns(4,5)P_2_ although it also displays significant activity *in vitro* toward PtdIns(3,4,5)P_3_ 39,40. Cell lines and zebrafish embryos deficient in OCRL1 display elevated PtdIns(4,5)P_2_ levels, indicating that this is a relevant *in vivo* substrate 41–44.

Following the catalytic domain is the ASH (ASPM, SPD2 and Hydin) domain, a domain with an immunoglobulin-like fold that is found in many proteins localized near cilia and centrosomes 45. The ASH domain of OCRL1 binds to members of the Rab GTPase family, which is important for the subcellular targeting of OCRL1 46. Interestingly, OCRL1 binds to Rabs in a manner atypical for effector proteins, which may explain the ability of OCRL1 to interact with many members of the Rab family 47. The RhoGAP-like domain is localized at the C-terminal end of the protein, directly adjacent to the ASH domain. This domain is catalytically inactive, but is able to bind to Rac1 and Cdc42, which may help localize OCRL1 to sites of actin assembly 48–50. Additionally, a conserved region within the RhoGAP-like domain binds to the F&H motif of the endocytic adaptor proteins APPL1 (adaptor protein containing pleckstrin-homology domain, PTB phosphotyrosine-binding domain and leucine zipper/bin-amphiphysin-rvs domain 1) and IPIP27A and B (inositol polyphosphate phosphatase-interacting protein of 27 KDa, also known as Ses1 and 2), which links OCRL1 to endocytic signaling and trafficking 50–53. As in the PH domain, there is a loop that extends outside the RhoGAP-like fold containing a clathrin-binding motif 38,50,54. As a result of alternative splicing, the region directly adjacent to the unstructured loop is different in the two known isoforms of OCRL1 55. OCRL1 isoform a has a longer loop, leading to better accessibility to clathrin and higher affinity clathrin binding than isoform b, which has a shorter loop 37.

## Cellular Functions of OCRL1

**Figure 2:**
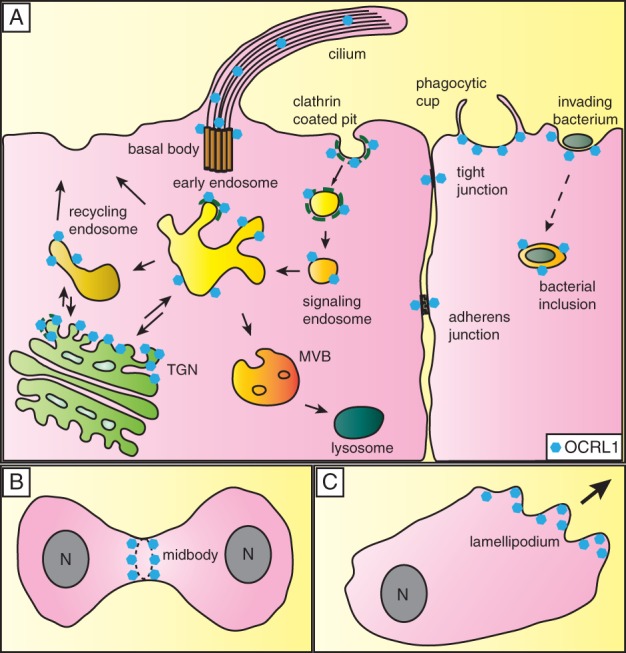
Cellular localization of OCRL1. A) OCRL1 (blue hexagons) has been localized to a number of cellular compartments. It is present at the TGN and various compartments of the endocytic pathway, where it resides at late-stage clathrin-coated pits, clathrin-coated vesicles, signaling endosomes, early or sorting endosomes and on recycling endosomes. OCRL1 has also been localized to the basal body of primary cilia, and it may also localize to the cilium itself. In maturing epithelia, OCRL1 transiently localizes to adherens and tight junctions. OCRL1 is recruited to phagosomes at a late stage in their formation, and is important for closure of the phagocytic cup as well as signaling events that occur post-sealing. OCRL1 is recruited to phagosomes generated by invading pathogenic bacteria such as *Yersinia* or *Listeria*, and has also been localized on intracellular inclusions generated by certain bacteria, e.g. *Legionella* or *Chlamydia*. B) OCRL1 localizes to the midbody in cells undergoing cytokinesis. C) OCRL1 has been localized to the lamellipodia of migrating fibroblasts.

**Figure 3:**
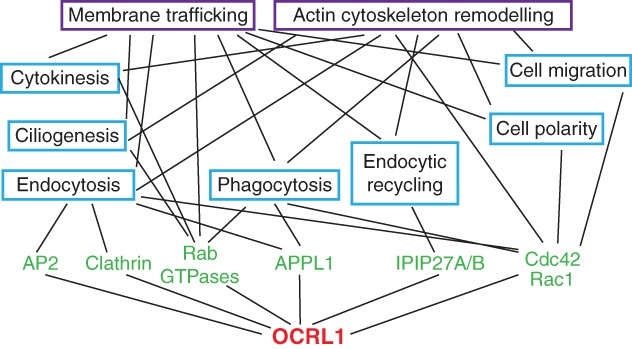
Network diagram showing the cellular functions of OCRL1. OCRL1 interaction partners are shown in green, and associated functions are indicated in blue boxes. Purple boxes highlight the two universal processes that are influenced by OCRL1, namely membrane trafficking and actin cytoskeleton remodeling. Both are relevant for all the functions shown in blue, and are linked accordingly. Note the diagram is not exhaustive, and some of the OCRL1-binding partners are likely to participate in several of the processes indicated. Although endocytosis and endocytic recycling are types of membrane trafficking, they are also shown separately as distinct processes given their reliance on different OCRL1-binding proteins.

OCRL1 is localized to several cellular compartments and has been implicated in a number of processes, which are described below (Figure 2; see also Figure 3).

### Membrane trafficking

Most available evidence supports a role for OCRL1 in membrane trafficking 36,37,50,52,54,56–58. OCRL1 is localized to early and recycling endosomes 38,50,52,54, and several studies have shown defective trafficking from endosomes to the *trans*-Golgi network (TGN) in OCRL1-deficient cells, or in cells expressing a dominant negative OCRL1 construct 54,56–58. Defective receptor recycling to the plasma membrane and sorting of mitogenic receptors to lysosomes has also been reported in OCRL1-deficient cells 57. These receptors were instead retained in enlarged early endosomes that had excessive amounts of PtdIns(4,5)P_2_ and actin on their cytoplasmic face 57. PtdIns(4,5)P_2_ promotes endosomal actin accumulation in two ways: activation of the nucleation-promoting factor N-WASP and inhibition of the actin-severing protein cofilin 9,57,59. Phosphorylation of cofilin, which also inhibits actin severing, has also been observed in OCRL1-deficient cells, suggesting a second, indirect, mechanism for cofilin inactivation 58. OCRL1 therefore appears to regulate or limit the assembly of actin on endosomal membranes. Endosomal actin plays an important role in sculpting membranes during the formation of trafficking intermediates that mediate recycling to the TGN and plasma membrane 60. We may therefore predict that OCRL1 regulates this process, in which case we can think of OCRL1 as constituting part of the trafficking machinery. However, an alternative viewpoint is that OCRL1 functions to maintain endosomal homeostasis, preventing ectopic accumulation of PtdIns(4,5)P_2_, rather than directly contributing to trafficking *per se*. Further studies will be required to make the distinction between these two possibilities.

OCRL1 directly interacts with clathrin heavy chain and the AP2 clathrin adaptor, and is recruited to clathrin-coated pits 36–38,50,54. OCRL1 recruitment occurs at a late stage in the vesicle formation process, and coincides with release of actin and actin-associated proteins required for carrier morphogenesis 50,61. These observations implicate OCRL1 in a late stage of vesicle biogenesis, possibly removal of the actin scaffold to allow release and movement of a newly formed vesicle into the cell. Although overexpression of mutant OCRL1 can perturb endocytic uptake 37, several studies have failed to detect any effects of OCRL1 depletion upon this process 56–58. As two other inositol 5-phosphatases, synaptojanin and SHIP2, are also present in clathrin-coated pits, OCRL1 may simply be redundant there. However, one should bear in mind that studies to date have only been carried out in tissue culture cells. It remains possible that cells with a higher rate of endocytic flux, such as neurons, will have a greater dependency upon OCRL1-mediated PtdIns(4,5)P_2_ hydrolysis. A third possibility is simply that OCRL1, although recruited into clathrin-coated pits, has no function there. It may be recruited at this early stage to allow delivery to the downstream endocytic compartments where it functions.

OCRL1 is also abundant at the TGN, and has been detected in clathrin-coated buds on this compartment 54,62. Again, the extent to which OCRL1 participates in trafficking from the TGN to the plasma membrane or endosomal compartments remains to be determined. One study reported reduced secretory trafficking of the TRPV6 calcium channel in OCRL1-deficient cells, but the exact steps affected and underlying mechanisms remain obscure 63.

### Phagocytosis

OCRL1 plays an important role in phagocytosis. This was first observed in *Dictyostelium discoideum*, where mutation of the OCRL1 homolog Dd5P4 leads to a failure to close the phagocytic cup 64. A role for OCRL1 in phagocytosis in mammalian cells has subsequently been shown 65,66. OCRL1 is recruited to phagosomes and catalyzes removal of phagosomal PtdIns(4,5)P_2_ at the closure stage. This helps remodel actin for phagosome closure as well as terminating synthesis of PtdIns(3,4,5)P_3_, thereby attenuating downstream Akt signaling. OCRL1 can be delivered to phagosomes in TGN- or endosome-derived vesicles, and is retained there through binding to APPL1 at the phagosome membrane 65,66.

OCRL1 plays an important role during infection by the pathogenic bacteria *Yersinia pseudotuberculosis* and *Listeria monocytogenes*, responsible for Far East scarlet-like fever and listeriosis, respectively 67,68. In both cases, OCRL1 recruitment to the invading bacterial phagosome coincides with removal of PtdIns(4,5)P_2_ and dissociation of bound actin, which is required for closure of the bacterial inclusion. Given its demonstrated importance in phagocytosis in studies to date, it is likely that other bacterial pathogens will exploit OCRL1 during their phagocytic entry into cells. Interestingly, OCRL1 also appears to function at the post-entry stage during infection by *Chlamydia* and *Legionella* 69,70. It localizes to the surface of bacterial inclusions formed by these organisms, and helps maintain the correct phosphoinositide composition for replication within the inclusion.

### Cell adhesion and migration

OCRL1 has been localized to lamellipodia, consistent with a possible role in cell migration 71,72. This was shown to be the case, as OCRL1-deficient Lowe syndrome fibroblasts or cells depleted of OCRL1 using RNA interference migrate poorly *in vitro* 72. Cell adhesion was also found to be defective in OCRL1-deficient cells 72. Loss of OCRL1 could perturb cell adhesion and migration in several ways, but the most likely mechanism is through dysregulation of the actin cytoskeleton. Indeed, levels of active Rac1 and cofilin, both important for actin dynamics within lamellipodia, are decreased in OCRL1-deficient cells 58. These effects could be direct, via modulation of PtdIns(4,5)P_2_ within lamellipodia that in turn impacts upon Rac1 activation and cofilin inactivation, or may arise indirectly through defective endocytic trafficking. Because Rac1 activation can occur on endosomes 73, defective endocytic cycling of Rac1 or its GEF could account for reduced Rac1 activation in OCRL1-deficient cells. This would be consistent with the observation that binding of OCRL1 to clathrin and AP2 is required for its function in cell migration 72. Further studies will be required to decipher the precise mechanisms involved.

### Cell polarity

The tissues affected in Lowe syndrome and Dent-2 disease comprise polarized cells, prompting the investigation of OCRL1 involvement in cell polarity. OCRL1 can transiently localize to adherens and tight junctions during establishment of cell polarity 74, and loss of OCRL1 leads to a failure to polarize effectively in both 2D and 3D culture 74,75. This could reflect a direct effect upon the localized assembly of junctional components, or a more indirect effect downstream from altered actin dynamics or endocytic trafficking. We favor the latter hypothesis, with defective endocytic recycling of junctional proteins leading to a failure to establish cell contacts and apicobasal polarity. Again, further studies are required to distinguish between these possibilities. It will also be important to determine the degree to which polarity of renal, lens and neuronal cells is affected in the disease state.

### Ciliogenesis

Several recent studies have found that OCRL1 plays a role in the biogenesis of cilia 75–77. Two studies reported fewer and shorter primary cilia upon loss of OCRL1 76,77, whereas a third study reported longer cilia 75. One study reported that OCRL1 was present within the primary cilium 77, whereas another found OCRL1 at the base of the cilium, near the basal body, but not within the cilium itself 76. Regardless of these differences, it would appear that OCRL1 is important for ciliogenesis, leading to the suggestion that Lowe syndrome is a type of ciliopathy 75–77. However, it should be noted that even though ciliopathies have a broad phenotypic spectrum 78, the most common manifestations are distinct from those seen in Lowe syndrome (discussed in greater detail below) 79.

How might OCRL1 regulate ciliogenesis? The most likely mechanism is by modulating trafficking of ciliary components into the cilium. Indeed, in cells depleted of OCRL1, trafficking of membrane marker proteins into the cilium is impaired 76. OCRL1 binds to Rab8, which is a key factor in polarized secretory trafficking into the cilium 46,47. Rab binding is important for OCRL1 function in ciliogenesis. IPIP27A, which links OCRL1 to endocytic trafficking, also appears to function in ciliogenesis 76. These observations suggest a role for OCRL1 in regulating trafficking into the cilium from both the secretory and endocytic pathways.

### Cytokinesis

Cytokinesis defects have been observed in *Drosophila* and mammalian cell lines lacking OCRL1 80,81. The phenotype is highly penetrant in *Drosophila* cells, with abortive furrow ingression leading to division failure and accumulation of binucleated cells 80. Interestingly, PtdIns(4,5)P_2_, which is typically enriched at the cleavage furrow and midbody, accumulates on enlarged endosomes in OCRL1-depleted cells, leading to the endosomal mistargeting of actin and associated cytokinesis machinery. Dysregulation of PtdIns(4,5)P_2_ and actin can therefore explain the observed cytokinesis failure in *Drosophila* cells. Similarly, although the cytokinesis defect is more subtle in OCRL1-depleted mammalian cells, dysregulation of the actin machinery also occurs 81. In this case, there is a failure to remove PtdIns(4,5)P_2_ from the midbody, which is required for actin remodeling during the final abscission step. Hence, cells stall at abscission and fail to complete cytokinesis. OCRL1 is recruited to the midbody by binding to Rab35, suggesting a direct role in modulating the pools of PtdIns(4,5)P_2_ and actin relevant for completion of abscission 81.

### Intracellular signaling

APPL1 is a Rab5 effector that functions as an endocytic scaffolding and signaling adaptor 82. APPL1 is localized to a subset of early endosomes, sometimes referred to as signaling endosomes, and participates in numerous signaling pathways. It interacts with many receptor proteins and notably can bind directly to Akt and modulate signaling from this kinase, which is important for cell survival, growth and proliferation. The interaction of OCRL1 with APPL1 therefore implicates OCRL1 in endocytic signaling 50. This could occur in a number of ways. OCRL1 could physically influence the binding of APPL1 to Akt, but this would seem unlikely. Alternatively, binding to APPL1 may help localize OCRL1 to sites of signaling, where it could attenuate Akt activation by reducing PtdIns(3,4,5)P_3_ levels, either through direct hydrolysis or by removal of PtdIns(4,5)P_2_, the precursor of PtdIns(3,4,5)P_3_ in mitogenic signaling. The latter has been observed in signaling that occurs during phagocytosis 65. In contrast, OCRL1-deficient zebrafish embryos have decreased levels of active Akt 43, suggesting that OCRL1 can influence signaling outputs in different ways, depending on the context. It is worth noting that dephosphorylation of PtdIns(3,4,5)P_3_ by OCRL1 will generate PtdIns(3,4)P_2_. Endosomal PtdIns(3,4)P_2_ has been shown to be important for Akt signaling and cell survival and proliferation 83,84. Hence, loss of OCRL1 could also impact upon Akt signaling by affecting the levels of endosomal PtdIns(3,4)P_2_.

OCRL1 may also influence signaling through modulation of intracellular calcium. Indeed, altered calcium signaling has been seen in OCRL1-deficient cells 75,85. This could be a direct effect, with elevated PtdIns(4,5)P_2_ leading to increased IP_3_ production (via phospholipase C-mediated hydrolysis) and mobilization of intracellular calcium. Alternatively, OCRL1 could influence calcium signaling, as well as other signaling pathways, through its effects upon ciliogenesis (cilia are key sites for the transduction of many signaling pathways). Further studies will be required to determine the extent to which OCRL1 may influence calcium signaling and the mechanisms involved.

### Common themes

At first glance, OCRL1 seems to participate in a myriad of cellular processes that are distinct from one another. However, closer analysis suggests that there may be a common underlying mechanism (see Figure 3). Most of the processes affected by loss of OCRL1 are actin dependent. The substrates of OCRL1, PtdIns(4,5)P_2_ and PtdIns(3,4,5)P_3_, strongly promote actin assembly 86. Hence, OCRL1 may influence the processes described above by regulating actin dynamics. Indeed, it has been known for several years that OCRL1-deficient cells have reduced numbers of stress fibers and increased punctate actin staining 87. Aberrant actin assembly has been observed on endosomes, phagosomes and at the midbody in OCRL1-deficient cells, which can explain the observed defects in endocytic trafficking 57, phagocytosis 65,66 and cytokinesis 80,81, respectively. As OCRL1 is also localized to lamellipodia, cellular junctions and may also be at the cilium or basal body, it could in principle directly regulate actin turnover at these locations to influence cell migration, adhesion, polarity and ciliogenesis 71,72,74,76,77. However, an alternative viewpoint is that OCRL1 indirectly affects the various processes, with a primary defect in endocytic trafficking leading to downstream consequences upon these other processes. This model is attractive in that all these processes are dependent upon endocytic trafficking. Hence, defective trafficking will have consequences upon phagocytosis, cytokinesis, cell adhesion and migration, cell polarization and ciliogenesis. It is important to remember, however, that the two models are not mutually exclusive, and it is possible that OCRL1 can affect various processes in both a direct and an indirect manner, either through localized actin remodeling and/or endocytic trafficking. Given the widespread localization of OCRL1, it remains possible that it will also influence other, as yet unrealized, actin-dependent processes within the cell.

### Phosphoinositide homeostasis

Cells lacking OCRL1 have elevated levels of PtdIns(4,5)P_2_ despite the presence of other inositol 5-phosphatases 41–44. Hence, it has been suggested that OCRL1 acts in a housekeeping capacity, preventing ectopic accumulation of PtdIns(4,5)P_2_ [and possibly PtdIns(3,4,5)P_3_] on intracellular membranes to help maintain phosphoinositide homeostasis within the cell 88. In support of this model, ectopic accumulation of PtdIns(4,5)P_2_ on endosomes has been observed in OCRL1-deficient mammalian and *Drosophila* cells 57,80. Thus, loss of OCRL1 could impact upon the processes described above through disruption of phosphoinositide homeostasis, leading to loss of compartment identity and dysregulation of downstream processes. Importantly, this model implies a more generalized, non-specific disruption of cellular function, as opposed to the specific regulation of spatially distinct, physiologically relevant pools of PtdIns(4,5)P_2_ [and possibly PtdIns(3,4,5)P_3_], as mentioned in the above section. Of course, the two possibilities are not mutually exclusive, and a role for OCRL1 both in regulating distinct functional phosphoinositide pools in addition to having a more general function in maintaining phosphoinositide homeostasis is possible.

It remains unclear where the ectopically accumulated endosomal PtdIns(4,5)P_2_ seen upon OCRL1 deficiency comes from. It may be delivered from the plasma membrane by endocytic vesicles or, conversely, generated *de novo* at the endosomal membrane. The latter hypothesis is somewhat contentious, but the recent identification of an endosomally localized PtdIns4P 5-kinase indicates that synthesis of PtdIns(4,5)P_2_ can occur on endosomes 89. It will therefore be interesting to determine the extent to which PtdIns(4,5)P_2_ synthesis takes place not only at the endosome but also at other endomembrane compartments within the cell.

## Analysis of OCRL1 in Animal Models

OCRL1 is almost ubiquitously expressed in humans, absent only from cells of hematopoietic origin 90. It is not entirely clear why only certain tissues are affected by loss of function mutations in OCRL1, but a major factor is INPP5B, a related inositol 5-phosphatase 23. INPP5B has a similar substrate preference to OCRL1, the same domain organization, overlapping subcellular distribution and shared interaction partners with OCRL1, although it does not bind clathrin or AP2 50,91. OCRL1 and INPP5B are the only human 5-phosphatases with a RhoGAP-like domain. All vertebrates appear to have both OCRL1 and INPP5B, whereas ‘lower eukaryotes’ such as *D. discoideum*, *Drosophila melanogaster* and *Caenorhabditis elegans* have only a single homolog with a RhoGAP-like domain 64,88. INPP5B is also widely expressed in human tissues 92, and, based on the similarities it has with OCRL1, it is reasonable to propose that INPP5B can compensate for loss of OCRL1, i.e. these proteins are functionally redundant in most cells within the body. However, another explanation that is not mutually exclusive is that the cellular processes dependent upon OCRL1 are of most importance to the cell types affected in Lowe syndrome and Dent-2 disease. In order to determine the extent to which these two possibilities can explain the nature of the symptoms seen in Lowe syndrome and Dent-2 disease, and to define the underlying pathophysiological mechanisms, several animal models have been developed. These are described below.

### Mouse

A knockout mouse for OCRL1 was generated in 1998, but surprisingly failed to recapitulate the symptoms seen in human Lowe syndrome and Dent-2 disease 92. Knockout of INPP5B in mice also has little effect (apart from a male sterility defect), but when both genes are knocked out, the result is early embryonic lethality 92. This observation strongly supports the notion that OCRL1 and INPP5B can functionally compensate for one another *in vivo*. Why the degree of compensation is greater in mice than humans is unclear, but appears to be due to two factors: higher expression levels of mouse INPP5B in the tissues affected in humans, and unusual splicing of the mouse INPP5B gene resulting in a slightly longer version of the protein 92,93. Although the effect of this murine-specific splicing, which results in an extended N-terminal linker domain, upon INPP5B function is unclear, confirmation of its importance *in vivo* has come from analysis of a ‘humanized’ mouse strain expressing human INPP5B in a double murine OCRL1/INPP5B knockout background 94. This mouse displays reduced growth and a renal tubulopathy similar to that seen in humans, with low-molecular-weight proteinuria and aminoaciduria. Therefore, it represents a good model to investigate the mechanisms underlying the renal dysfunction in Lowe syndrome and Dent-2 disease. Interestingly, the mouse does not display ocular or neurological defects, which may be a consequence of the way it was generated, with BAC-driven expression of human INPP5B resulting in a 5- to 10-fold greater expression level than that of the endogenous murine INPP5B 94. It will be interesting to observe the phenotype of a mouse expressing human INPP5B at a more physiological level.

### *Zebrafish* (Danio rerio)

OCRL1 and INPP5B are well conserved in zebrafish in terms of sequence, domain organization and the presence of the known protein interaction motifs 43. The tissue-specific splicing of OCRL1 is also conserved in zebrafish, with isoform a, which has better clathrin-binding ability 37, expressed in all tissues, and isoform b expressed in all tissues apart from the brain, as seen in humans 43. Both OCRL1 and INPP5B are expressed during embryogenesis, with maternal and zygotic transcripts observed in the initial stages of embryonic development (43, Oltrabella et al., unpublished data). This implies a role for these proteins in early development. A mutant zebrafish in which OCRL1 expression is attenuated by insertion of a retrovirus in the promoter region has been generated 43. This mutant displays neurodevelopmental defects including delayed brain and eye development, with reduced cell proliferation and increased apoptosis observed in the neural tissue. The OCRL1 mutant also exhibits an increased susceptibility to febrile seizures, and has cystic lesions in the brain, with accompanying gliosis, both of which have been observed in Lowe syndrome patients 33. The mechanisms responsible for these neurological manifestations remain to be determined, but rescue experiments in the mutant line indicate an important role for clathrin binding, suggesting that defective clathrin-mediated trafficking is responsible 43. This could impact upon presynaptic function through perturbation of synaptic vesicle recycling, or postsynaptic function through altered trafficking of neurotransmitter or growth factor receptors or their downstream signaling components, which are essential for neuronal survival and function. Evidence for the latter comes from the reduced Akt signaling seen in the OCRL1 mutant 43. An alternative and not mutually exclusive explanation for the neurodevelopmental defects is the defective biogenesis or maintenance of cilia in the OCRL1 mutant.

Three independent studies have indicated a role for OCRL1 in ciliogenesis during zebrafish development 75–77. These studies used depletion of OCRL1 by injection of antisense morpholinos 75–77, with one study also using the OCRL1 mutant described above 76. In two cases cilia were fewer and shorter 76,77, whereas one study reported longer cilia 75. In all cases there were morphological defects consistent with defective cilia, namely a curved body axis, smaller head and eyes, craniofacial malformation, reduced pigmentation, reversed heart looping and cystic pronephros (embryonic kidneys). Based on these observations, and because the organs affected in Lowe syndrome and Dent-2 disease are similar to those affected in ciliopathies (brain, eyes and kidneys), it has been suggested that Lowe syndrome and Dent-2 disease are a type of ciliopathy 75–77. However, the ocular and renal symptoms of Lowe syndrome and Dent-2 disease (cataracts, glaucoma and renal tubulopathy) are not typically observed in ciliopathies, which usually result in retinopathy and renal cysts, as well as additional defects not seen in Lowe syndrome and Dent-2 patients (liver disease, polydactyly and situs inversus) 78. Hence, it is probably oversimplistic to classify Lowe syndrome and Dent-2 as ciliopathies. Nevertheless, it is likely that loss of cilia function contributes to some of the pathophysiological manifestations of these disorders.

Clearly INPP5B cannot fully compensate for loss of OCRL1 in zebrafish, because depletion of OCRL1 alone results in a phenotype. However, there does appear to be overlapping functionality because morpholino-induced depletion of INPP5B also results in a ciliogenesis defect, with a phenotype similar to that seen in OCRL1 morphants 95. Overexpression of INPP5B can also partially restore the ciliogenesis defect of OCRL1 morphants, supporting the idea that both proteins function in ciliogenesis 76. This raises the question of why loss of either protein gives a phenotype. One possibility is that OCRL1 and INPP5B are required for ciliogenesis in different cells, consistent with the apparent localization of the proteins to distinct cell types in the eye 95.

**Figure 4:**
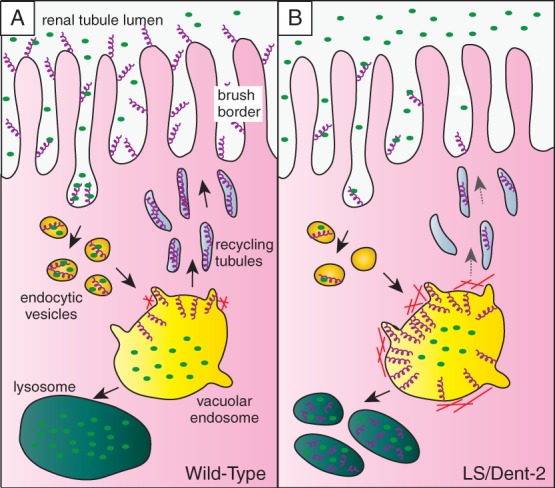
Model for OCRL1 function in endocytic trafficking of megalin in the renal tubule. A) The multiligand receptor megalin (purple helices) is abundant at the apical membrane of the epithelial cells lining the proximal tubule, where it binds to low-molecular-weight proteins in the renal filtrate (green ovals). Megalin is internalized by endocytosis and delivered via endocytic vesicles to the large vacuolar sorting endosomes found in this cell type. The low pH in the vacuolar endosome dissociates the megalin ligands, which in most cases are delivered to the lysosome for degradation. Megalin is sorted into recycling tubules that bud from the vacuolar endosome and deliver the receptor back to the plasma membrane for further rounds of endocytosis and recycling. B) Upon OCRL1 deficiency, megalin trafficking is impaired. Recycling of megalin from vacuolar early endosomes to the plasma membrane occurs less efficiently owing to aberrant accumulation of actin at the endosomal membrane. This results in endosomal accumulation of the receptor and missorting to the lysosome. The reduced abundance of megalin at the plasma membrane is responsible for reduced endocytosis of low-molecular-weight proteins from the renal filtrate, explaining the low-molecular-weight proteinuria seen in Lowe syndrome and Dent-2 disease.

It has been proposed that defective endocytic trafficking can account for the renal tubulopathy seen in Lowe syndrome and Dent-2 disease 96. The multiligand receptor megalin (also called LRP2) is abundant at the apical pole of the proximal tubule and mediates the retrieval of numerous low-molecular-weight proteins from the renal filtrate 97 (Figure 4). Mutation of megalin in humans causes Donnai–Barrow syndrome, which is characterized by low-molecular-weight proteinuria similar to that seen in Lowe syndrome and Dent-2 disease 98. In both megalin-deficient mice and zebrafish there is a profound apical endocytic defect in the proximal tubular cells 99–101. In Dent-1 disease, which has the same renal pathology as Lowe syndrome and Dent-2, there is also an endocytic defect 102–104. Dent-1 disease is caused by mutation of the endosomal chloride/proton antiporter CLC5, which helps maintain endosomal pH and chloride levels 30,102–105. Knockout of CLC5 in mice causes impaired recycling of megalin from endosomes to the plasma membrane, explaining the failure to internalize proteins from the renal filtrate 102,103.

Defective endocytic trafficking of megalin could therefore explain the low-molecular-weight proteinuria in Lowe syndrome and Dent-2 disease (Figure 4). Studies in tissue culture cells are consistent with this hypothesis 57, and recent work in our laboratory using OCRL1 mutant zebrafish has shown that OCRL1 does indeed play a role in endocytic trafficking within the renal tubule (Pietka et al., unpublished data). There is reduced endocytosis from the renal filtrate in OCRL1 mutant embryos, and an accompanying mislocalization of megalin within the endocytic pathway. These defects are independent of effects upon cilia, indicating that it is the endocytic function of OCRL1 that is important for the renal tubulopathy phenotype. The mechanisms underlying the endocytic defect within renal tubules remain to be determined, but dysregulation of endosomal actin resulting in impaired receptor recycling may be responsible, as shown *in vitro* 57.

### Dictyostelium discoideum

The slime mold *D. discoideum* contains a single OCRL1/INPP5B homolog called Dd5P4 64. Deletion of this gene results in a defect in phagocytosis 64. Whether other cellular processes are also affected in this organism remains to be determined. It is interesting to note that *D. discoideum* does not appear to have homologs of the two known F&H domain proteins APPL1 and IPIP27, yet the binding interface for the F&H motif is conserved in Dd5P4 53. This suggests that additional F&H domain proteins exist in this species that may also be conserved in higher eukaryotes.

### Other models

Although the established model organisms *D. melanogaster* and *C. elegans* both contain a single OCRL1/INPP5B homolog 64,88, they have so far not been used to explore the *in vivo* functions of this protein. However, the presence of only a single homolog is likely to prevent the redundancy that occurs in vertebrate models, making these attractive model organisms for such analysis. Future studies in these organisms are therefore likely to yield important information on the *in vivo* functions of both OCRL1 and INPP5B. Studies in other eukaryotes with a single OCRL1/INPP5B homolog are also likely to prove informative. For example, the *Trypanosoma brucei* OCRL1 homolog (TbOCRL) is the only RhoGAP-like protein in this organism, pointing to an important evolutionary conserved function for the protein 106.

## A Single Mechanism for Lowe Syndrome and Dent-2 Disease?

The wide array of interaction partners for OCRL1 and the multitude of cellular functions affected by loss of OCRL1 function make elucidating the mechanisms underlying Lowe syndrome and Dent-2 disease a complex task. This is further compounded by the redundancy with INPP5B and the likelihood of other so far unknown genetic ‘modifiers’ that impact upon the severity of the phenotype. Defects in cilia formation and function are likely to contribute to the pathophysiological manifestations of Lowe syndrome and Dent-2 disease, but, as described above, are unlikely to be the sole cause. Defective endocytic trafficking is also likely to contribute to the phenotype, certainly in the proximal tubule. It is interesting that the cell types affected in Lowe syndrome and Dent-2 disease (neurons, proximal tubular cells and lens epithelial cells) have high intrinsic rates of endocytosis, which may make them more sensitive to loss of OCRL1 function. Conversely, these cell types are also polarized, suggesting that defective cell polarity is important, although evidence for this *in vivo* is currently lacking. However, there are many other cell types in the body that have high endocytic rates or that are polarized, which remain unaffected in Lowe syndrome and Dent-2 disease. Hence, it is likely a combination of factors that determine the Lowe syndrome and Dent-2 phenotype. These include redundancy with INPP5B, which is likely to vary between cells, and the particular requirement of cell types in the body upon the various cellular processes that are affected by loss of OCRL1. As loss of OCRL1 affects a number of cellular processes, it is possible that rather than having a single underlying cellular basis, the disease phenotype reflects a manifestation of several cellular defects. A careful approach will be required to properly address this issue, combining cell biology with studies in animal models.

## Conclusions and Future Directions

Studies on OCRL1 indicate that a loss of a single phosphoinositide-metabolizing enzyme can impact upon a large number of cellular processes. Moreover, studies in animal models indicate that loss of OCRL1 can affect different tissues in apparently different ways. Thus, although Lowe syndrome and Dent-2 disease are monogenic disorders, deciphering the disease mechanisms is far from trivial. Nevertheless, through the combination of careful cell-based experiments combined with the generation of animal models significant progress has been made. It is likely that the continued application of both approaches will lead to new discoveries relevant not only to OCRL1 and Lowe syndrome/Dent-2 disease but also our understanding of cellular and organismal physiology as a whole.

Our improved understanding of OCRL1 biology and the development of animal models should lead to the better design of and improved screening for potential therapeutics to treat Lowe syndrome and Dent disease. A recent example of a rationally designed compound is provided by PHDM (small-molecule PH domain mimetic), which can sequester cellular PtdIns(4,5)P_2_ and attenuate PtdIns(4,5)P_2_-dependent processes such as endocytosis and actin dynamics 107. The further development of this compound and other compounds that influence cellular PtdIns(4,5)P_2_ levels may prove a useful strategy to develop new therapeutics. Moreover, the ability to screen compound libraries both at the cellular and organismal level should prove beneficial, providing that robust and specific assays for high-throughput analysis of OCRL1 function can be developed 108,109. The development of such assays should pave the way for identification of novel potential treatments for both Lowe syndrome and Dent-2 disease.
